# Impact of the 2015/2016 El Niño on the terrestrial carbon cycle constrained by bottom-up and top-down approaches

**DOI:** 10.1098/rstb.2017.0304

**Published:** 2018-10-08

**Authors:** Ana Bastos, Pierre Friedlingstein, Stephen Sitch, Chi Chen, Arnaud Mialon, Jean-Pierre Wigneron, Vivek K. Arora, Peter R. Briggs, Josep G. Canadell, Philippe Ciais, Frédéric Chevallier, Lei Cheng, Christine Delire, Vanessa Haverd, Atul K. Jain, Fortunat Joos, Etsushi Kato, Sebastian Lienert, Danica Lombardozzi, Joe R. Melton, Ranga Myneni, Julia E. M. S. Nabel, Julia Pongratz, Benjamin Poulter, Christian Rödenbeck, Roland Séférian, Hanqin Tian, Christel van Eck, Nicolas Viovy, Nicolas Vuichard, Anthony P. Walker, Andy Wiltshire, Jia Yang, Sönke Zaehle, Ning Zeng, Dan Zhu

**Affiliations:** 1 Department of Geography, Ludwig Maximilians University Munich, Luisenstr. 37, Munich D-80333, Germany; 2 Laboratoire des Sciences du Climat et de l'Environnement (LSCE), CEA-CNRS-UVSQ, UMR8212, Gif-sur-Yvette 91191, France; 3 College of Engineering, Mathematics and Physical Sciences, University of Exeter, Exeter EX4 4QF, UK; 4 College of Life and Environmental Sciences, University of Exeter, Exeter EX4 4RJ, UK; 5 Department of Earth and Environment, Boston University, Boston, MA 02215, USA; 6 CESBIO, Université de Toulouse, CNES/CNRS/IRD/UPS, 31400 Toulouse, France; 7 UMR 1391 ISPA, INRA, Centre Bordeaux Aquitaine, Villenave d'Ornon 33883, France; 8 Canadian Centre for Climate Modelling and Analysis, Environment and Climate Change Canada, University of Victoria, Victoria, British Columbia, Canada V8W2Y2; 9 CSIRO Oceans and Atmosphere, Canberra, ACT 2601, Australia; 10 Global Carbon Project, CSIRO Oceans and Atmosphere, Canberra, ACT 2601, Australia; 11 State Key Laboratory of Water Resources and Hydropower Engineering Science, Wuhan University, Wuhan 430072, People's Republic of China; 12 Centre National de Recherches Météorologiques, CNRM, Unité 3589 CNRS/Meteo-France/Université Fédérale de Toulouse, Av G Coriolis, Toulouse 31057, France; 13 Department of Atmospheric Sciences, University of Illinois, Urbana, IL 61801, USA; 14 Climate and Environmental Physics, Physics Institute and Oeschger Centre for Climate Change Research, University of Bern, Bern CH-3012, Switzerland; 15 Institute of Applied Energy (IAE), Minato, Tokyo 105-0003, Japan; 16 Climate and Global Dynamics Division, National Center for Atmospheric Research, Boulder, CO 80302, USA; 17 Climate Processes Section, Environment and Climate Change Canada, Downsview, Ontario, Canada V8W2Y2; 18 Max Planck Institute for Meteorology, Hamburg 20146, Germany; 19 NASA Goddard Space Flight Center, Biospheric Sciences Lab, Greenbelt, MD 20816, USA; 20 Max Planck Institute for Biogeochemistry, 07745 Jena, Germany; 21 International Center for Climate and Global Change Research, School of Forestry and Wildlife Sciences, Auburn University, 602 Duncan Drive, Auburn, AL 36849, USA; 22 Department of Geoscience, Environment and Society, CP 160/02, Université Libre de Bruxelles, Brussels 1050, Belgium; 23 Environmental Sciences Division and Climate Change Science Institute, Oak Ridge National Laboratory, Oak Ridge, TN 37831, USA; 24 Met Office Hadley Centre, Exeter EX1 3PB, UK; 25 Department of Atmospheric and Oceanic Science and Earth System Science Interdisciplinary Center, University of Maryland, College Park, MD 100029, USA; 26 State Key Laboratory of Numerical Modelling for Atmospheric Sciences and Geophysical Fluid Dynamics, Institute of Atmospheric Physics, Beijing 20740, People's Republic of China

**Keywords:** carbon cycle, El Niño/Southern Oscillation, land-surface models, atmospheric inversions

## Abstract

Evaluating the response of the land carbon sink to the anomalies in temperature and drought imposed by El Niño events provides insights into the present-day carbon cycle and its climate-driven variability. It is also a necessary step to build confidence in terrestrial ecosystems models' response to the warming and drying stresses expected in the future over many continents, and particularly in the tropics. Here we present an in-depth analysis of the response of the terrestrial carbon cycle to the 2015/2016 El Niño that imposed extreme warming and dry conditions in the tropics and other sensitive regions. First, we provide a synthesis of the spatio-temporal evolution of anomalies in net land–atmosphere CO_2_ fluxes estimated by two *in situ* measurements based on atmospheric inversions and 16 land-surface models (LSMs) from TRENDYv6. Simulated changes in ecosystem productivity, decomposition rates and fire emissions are also investigated. Inversions and LSMs generally agree on the decrease and subsequent recovery of the land sink in response to the onset, peak and demise of El Niño conditions and point to the decreased strength of the land carbon sink: by 0.4–0.7 PgC yr^−1^ (inversions) and by 1.0 PgC yr^−1^ (LSMs) during 2015/2016. LSM simulations indicate that a decrease in productivity, rather than increase in respiration, dominated the net biome productivity anomalies in response to ENSO throughout the tropics, mainly associated with prolonged drought conditions.

This article is part of a discussion meeting issue ‘The impact of the 2015/2016 El Niño on the terrestrial tropical carbon cycle: patterns, mechanisms and implications’.

## 1. Introduction

The global terrestrial CO_2_ sink has increased steadily in the past decades but presents high year-to-year variations that, in turn, dominate inter-annual variability (IAV) in the atmospheric CO_2_ growth rate [1]. As the atmospheric CO_2_ growth rate is highly correlated with tropical temperature [2], IAV in the land sink has been mainly attributed to tropical forests [2], but semi-arid ecosystems appear to be increasingly important [3–5].

The El Niño/Southern Oscillation (ENSO) is an atmosphere–ocean variability pattern that drives temperature and rainfall variations in the tropics, with teleconnections that extend worldwide [6]. El Niño events strongly reduce the global land sink by up to 2PgC [7], leading to high atmospheric CO_2_ growth rates [1]. El Niño events promote drought conditions in the Amazon forest, leading to increased tree mortality and reduced carbon storage [8,9] and widespread fires, particularly in southeast Asia [10]. ENSO impacts extend beyond the tropics, controlling IAV in sub-tropical ecosystem productivity [11], especially water-limited ecosystems in the Southern Hemisphere [3,4,12]. Most Coupled Model Intercomparison Project Phase 5 (CMIP5) models projected a two-fold increase in the frequency of extreme El Niño events in the future decades [13], associated with intensification of ENSO-related anomalies in the carbon cycle [14]. However, nonlinear ENSO dynamics found in observations and one model might imply suppressed extreme El Niño events under warming [15].

Additionally, ENSO affects key regions and processes that are sources of uncertainty in future carbon cycle projections [3,16]. It is still unclear if temperature [2] or water-availability [3,9,11] drive ecosystems' response to ENSO, and how gross primary productivity (GPP) and terrestrial ecosystem respiration (TER) contribute to IAV. Analysis of model ensembles suggests that because water availability enhances both GPP and TER, its effects are cancelled out, and only the temperature signal emerges [2,5]. Jung *et al.* [5] also showed that water availability is the primary driver of carbon fluxes at the local scale, but anomalies tend to compensate spatially, so temperature emerges as a stronger driver with increasing spatial aggregation.

More generally, IAV in the carbon cycle is still not well understood, and neither data-driven models [17] nor Earth-System Models [18] capture its amplitude. In the 2017 Global Carbon Budget [1], land–atmosphere CO_2_ fluxes from land-surface models (LSMs, bottom-up) forced with observed climate and land-use change (LUC) show good agreement with estimates from atmospheric transport model inversions (top-down) for global totals but differ at regional or zonal scale [1]. The 2015/2016 El Niño is especially interesting, as 2015 registered record atmospheric CO_2_ growth rate in spite of widespread record-breaking greening and stabilization of fossil-fuel emissions [1,19]. The 2015/2016 El Niño therefore provides a good study case to understand the response of ecosystems to warm and dry extremes potentially concurrent with global vegetation greening.

The strong El Niño event started around May 2015 and persisted until mid-2016, being the strongest event since the 1950s [20]. Record-breaking temperatures and drought were registered in the Amazon from October 2015 onwards. The drought extent in the Amazon was comparable to 1997/1998 but the extreme temperatures led to an exacerbation of dryness, with extreme drought conditions affecting double the extent of 1997/1998 [20].

According to LeQuéré *et al.* [1], the atmospheric CO_2_ growth rate in 2015 and 2016 was 1.6 and 1.5 PgC yr^−1^ higher than during the 2011–2016 period, respectively, yet CO_2_ emissions from fossil fuel and LUC combined were only 0.2–0.4 PgC yr^−1^ above the previous 5-year mean. Ocean uptake was estimated to be slightly larger (0.2 PgC yr^−1^) in 2015/2016 than the 2010–2014 average. Table 1 shows the residual sink needed to close the global carbon budget: the terrestrial CO_2_ uptake had to be reduced by 1.4 PgC yr^−1^ in 2015 and by 1.5 PgC yr^−1^ in 2016. In the same period, but using the year of 2011 as a reference, Liu *et al.* [21] reported much higher losses of CO_2_ over the pan-tropical regions in 2015 alone (2.5 PgC). Contrary to the 1997/1998 event, the anomaly in the land sink during 2015/2016 does not appear to be associated with major fire emissions. Although the development of El Niño coincided with enhanced fire activity in Southeast Asia, fire emissions in the region were reported to be only half of the emissions during the previous El Niño in 1997/1998, following rainfall return in November 2015 [22]. GFED4.1s [23] reports fire emissions 0.3 PgC yr^−1^ higher than the previous 5 years in 2015, but lower by 0.1 PgC yr^−1^ in 2016 (table 1).

**Table 1.** Global carbon budget during 2015, 2016 from the latest Global Carbon Project global carbon budget estimates (GCB2017v1.2, [1]). Annual atmospheric CO_2_ growth rate (*G*_ATM_), fossil fuel and LUC emissions (*E*_FF_ and *E*_LUC_, respectively) and the total sinks partitioned into ocean and land fluxes. The numbers in brackets indicate the corresponding anomaly relative to the previous 5-year period. The land sink is estimated here as the residual from the global carbon budget (i.e. *E*_FF_ + *E*_LUC_ − *G*_ATM_ − *O*). Fire emission anomalies from GFED4.1s (1997–2016) are shown for comparison with the values in the terrestrial sink.

C budget (PgC yr^−1^)	*G*_ATM_	*E*_FF_	*E*_LUC_	sinks (ocean + land)	ocean	land	fire emissions
2010–2014	4.6	9.6	1.4	6.3	2.4	4.0	2.0
2015	6.2 (+1.6)	9.8 (+0.2)	1.5 (+0.1)	4.1 (−1.2)	2.6 (+0.2)	2.6 (−1.4)	2.3 (+0.3)
2016	6.1 (+1.5)	9.9 (+0.3)	1.3 (−0.1)	5.3 (−1.0)	2.6 (+0.2)	2.4 (−1.6)	1.9 (−0.1)

Here we quantify the response of the terrestrial carbon cycle to El Niño in 2015/2016 using multiple data-based and modelled datasets. We track the evolution of anomalies in the net land–atmosphere CO_2_ flux during the development and decline of the 2015/2016 El Niño estimated by two atmospheric transport model CO_2_ inversions [24,25] and compare them with the net terrestrial CO_2_ uptake and its component fluxes (gross primary productivity (GPP), total ecosystem respiration (TER), fire) simulated by 16 LSMs in the latest TRENDY intercomparison project (v6, table 2) [1,42]. We evaluate the consistency and robustness of carbon spatio-temporal dynamics between top-down and bottom-up approaches and compare the results from LSMs with anomalies with satellite-based datasets.

**Table 2.** LSMs used in this study. From the 16 LSMs used here, 14 contributed to the latest global carbon budget (GCB2017v1.2, [1]). All models followed the protocol of TRENDYv6 and are therefore included here.

model	GCB2017v1.2	monthly fire emissions	reference
CABLE	Y	N	[26]
CLASS-CTEM	Y	Y	[27]
CLM4.5(BGC)	Y	Y	[28]
DLEM	Y	N	[29]
ISAM	Y	N	[30]
JSBACH	Y	Y	[31]
JULES	Y	N	[32]
LPJ	Y	annual	[33]
LPX-Bern	Y	Y	[34]
OCN	Y	N	[35]
ORCHIDEE	Y	N	[36]
ORCHIDEE-MICT	Y	Y	[37]
SDGVM	Y	annual	[38]
SURFEX	N	Y	[39]
VEGAS	N	N	[40]
VISIT	Y	Y	[41]

## 2. Material and methods

### (a) Atmospheric CO_2_ inversion fluxes

Here we use three observation-based datasets of net land–atmosphere surface fluxes: the Copernicus Atmosphere Monitoring Service (CAMS) atmospheric inversion (henceforth simply ‘inversion’) version 16r1 [24,43], and the Jena CarboScope inversion (update of [25,44] compare with Rödenbeck *et al.* [45]) versions s76_v4.1 and s04_v4.1 (CarboScope76 and CarboScope04 henceforth). The inversions provide terrestrial (and oceanic) surface CO_2_ fluxes, CAMS weekly fluxes at 1.9°latitude × 3.75°longitude resolution, and CarboScope daily fluxes at 4°latitude × 5°longitude resolution. CAMS 16r1 uses 119 atmospheric stations over the different time frames for which they provide data, starting in 1979. CarboScope76 (CarboScope04) uses 10 (59) stations continuously available throughout 1976–2016 (2004–2016). All inversions are regularized by *a priori* information. CAMS uses climatological natural fluxes and time-varying ocean, wildfire and fossil-fuel fluxes with error correlation lengths of 4 weeks and 500 km (1000 km) over land (ocean) [46]. CarboScope uses a zero land prior, and *a priori* correlations of about 1600 km in longitude direction, 800 km in latitude direction and about 3 weeks. The inversions further differ in the transport model used, and other characteristics. Thus, they provide a range of uncertainty for observation-based top-down CO_2_ flux estimates [19]. We focus on the 38-year period from 1979 until 2016 and calculate monthly anomalies of net land–atmosphere fluxes by subtracting the mean seasonal cycle and the monthly long-term trend (using a simple linear fit). We aggregate the inversion results over large regions (global terrestrial surface and tropical band between 23°S and 23°N), as flux estimates from inversions carry smaller relative uncertainties on the larger spatial scale [47].

### (b) Land-surface models

LSMs simulate the key energy, hydrological and carbon cycle processes in ecosystems, allowing insights on the mechanisms controlling anomalies in land–atmosphere CO_2_ fluxes and their drivers. The TRENDY intercomparison project coordinated historical LSM simulations and compiled outputs of CO_2_ fluxes among other variables [42]. We use 16 LSMs from the latest TRENDYv6 simulations [1] (table 2), which provide monthly CO_2_ fluxes during 1860–2016. In TRENDYv6 S3 simulations, models are forced by historical data of (i) atmospheric CO_2_ concentrations, (ii) climate observations from CRU-NCEP v8 [48,49] and (iii) human-induced land-cover changes and management from the HYDE [50,51] and the Land-Use Harmonization LUH2 v2 h [52] datasets (extended to 2016 as described in [1]). We analyse monthly values of net biome productivity (NBP), GPP, total ecosystem respiration (TER) and fire emissions simulated by the models (only 7 models) and annual leaf-area index (LAI, 12 models). NBP corresponds to the simulated net atmosphere–land flux (positive sign for a CO_2_ sink) and is comparable to top-down estimates of net land–atmosphere CO_2_ fluxes, although the latter include lateral C fluxes (the land–ocean transport of C in freshwater and coastal areas and C fluxes due to trade/import export) [1,53] not simulated by the models. However, we focus on flux anomalies that should not be substantially affected by lateral fluxes because they are assumed to vary little between years. To produce a spatially consistent ensemble, model outputs were remapped to a common regular 1° × 1° grid. The model data were selected for the 38-year long period 1979–2016, common to inversions.

### (c) Satellite-based data

We compare anomalies from inversions and LSMs with two remote-sensing datasets that provide proxies for ecosystem activity and a satellite-based GPP product.

LAI is defined as the one-sided green leaf area per unit ground area in broadleaf canopies and as one-half of the green needle surface area in needleleaf canopies, which depicts the greenness of vegetation. We used Collection 6 Terra and Aqua MODIS LAI products (MOD15A2H and MYD15A2H) [54,55]. The original datasets were available as 8-day composites in 500 m sinusoidal projection. We checked the quality flags (clouds, aerosols, etc.) to get high-quality LAI as described by Samanta *et al*. [56]. The original data were re-projected onto a 1/12° × 1/12° grid by averaging the high-quality LAI. After that, the two LAI datasets were combined to bi-monthly time-steps by taking the mean of LAI values in each 8-day composite, weighted by the number of days that each 8-day composite locates in the specific half-month window. Finally, the annual average LAI and its anomaly relative to the record period (2000–2016) were calculated for each pixel. Anomalies in LAI reflect changes in the canopy leaf density and can therefore track plant stress response to drought.

Cheng *et al.* [57] used ground-based and remotely sensed land and atmospheric observations, combined with water use efficiency (WUE) model and evapotranspiration data from global land evaporation Amsterdam model (GLEAM), to calculate global annual GPP between 2000 and 2016 at 0.5 × 0.5° resolution. The WUE model was developed by upscaling leaf WUE directly and considers the controls of vapour pressure deficit and physiological functioning on WUE. The model has been derived independently from GPP and evapotranspiration data, and therefore, can be used to evaluate simulated GPP.

Vegetation optical depth (VOD) is an estimate of the vegetation extinction effects on microwave radiation and increases with increasing vegetation density, being therefore a good proxy of biomass [58]. Brandt *et al.* [59] have shown that the new L-band soil moisture and ocean salinity (SMOS) VOD (L-VOD) retrieved from the SMOS-IC algorithm (Version V105 [60]) relates almost linearly to biomass and is thus relevant to monitor carbon stocks at continental scales. In this algorithm, no auxiliary data (either from atmospheric models or remote sensing optical observations) are used, except for surface temperature data from European Centre for Medium-Range Weather Forecasts (see [58,60] for more details). As L-VOD shows a strong relationship with aboveground biomass stocks, the time-derivative of L-VOD can be directly related to variations in biomass, and thus comparable with the aboveground component of NBP.

## 3. Results

### (a) Global and tropical net biome productivity anomalies

**Figure 1.**
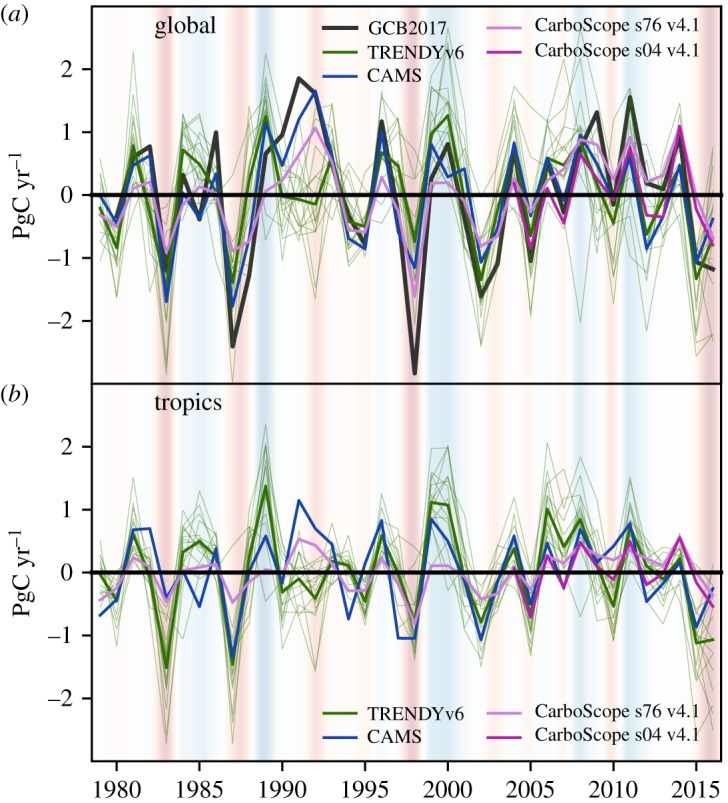
Time-series of detrended annual NBP_anom_ for the globe (*a*) and tropical regions (*b*), estimated as the residual sink by GCB2017 (black, globe only), CAMS v16r1 (blue), CarboScope76 (light magenta) and CarboScope04 (magenta) atmospheric inversions and TRENDYv6 models (green, thicker line indicates the multi-model ensemble mean (MMEM)). NBP_anom_ is defined as the net atmosphere-to-land CO_2_ flux: positive anomalies indicate stronger-than-average CO_2_ sinks or lower-than-average CO_2_ sources. The shades in the background of both panels show the ENSO states (red – El Niño and blue – La Niña).

Figure 1 compares annual global and tropical NBP from inversions and LSMs after removing the mean seasonal-cycle and linear trend during 1979–2016. Anomalies are indicated in subscript and positive values indicate enhanced atmosphere-to-land CO_2_ flux. We further compare the global NBP anomalies from inversions and LSMs with the anomalies of the residual land-sink from GCP2017.

The three datasets show consistent variability patterns over the 37-year period, but GCB2017 estimates stronger residual sink anomalies in certain years, e.g. 1991 (positive) or 1997 (negative). Although individual LSMs do not necessarily capture the main variability patterns of NBP reported by inversions, the multi-model ensemble mean (MMEM) is generally close to inversion values both globally and in the tropics. The exception in 1991/1992 is likely to be due to the response of the ecosystems to the variations in net direct and diffuse fraction of short-wave radiation following Mt. Pinatubo eruption [1,61], the latter not included in the TRENDY forcing.

Inversions and LSMs agree well in global NBP_anom_ during the two El Niño events in the 1980s (anomalies of ca. −1 to −2 PgC yr^−1^). In 1982, anomalies from inversions and LSMs are very close to the GCB2017 estimate, while in 1987 both approaches underestimate the negative anomaly (especially CarboScope76). In 1997/1998, inversions differ by up to 0.5 PgC yr^−1^ (1998), and some LSMs indicate a global sink anomaly, rather than a source anomaly. The MMEM average anomalies in 2015/2016 (−1.0 yr^−1^) are close to the GCB2017 residual sink anomalies (−1.1 PgC yr^−1^), while inversions point to weaker anomalies (−0.7 PgC yr^−1^ for CAMS, −0.4 PgC yr^−1^ for CarboScope76, −0.5 PgC yr^−1^ for CarboScope04). In the tropical band, LSMs agree better with inversions (CAMS and CarboScope04) for most ENSO events than at global scale, but estimate larger negative anomalies than inversions in 1983 and 2016. CarboScope76 shows too low variability and therefore we use CarboScope04 for the analysis of the 2015/2016 event.

### (b) Spatial net biome productivity anomalies in 2015/2016

**Figure 2.**
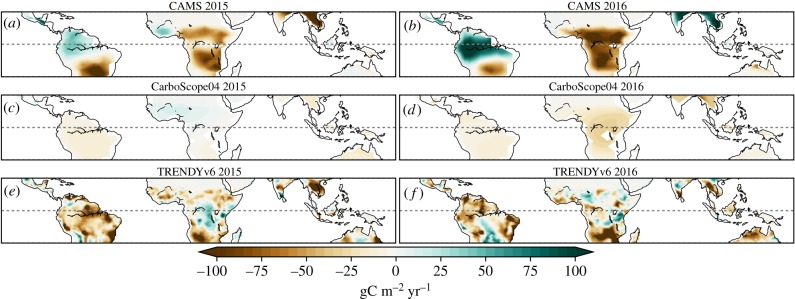
NBP_anom_ maps for the two recent El Niño years (2015/2016, (*a*,*c*,*e*)/(*b,d,f*)) estimated by CAMS (*a,b*) and CarboScope04 (*c,d*) inversions and the TRENDYv6 MMEM (*e,f*). Anomalies are calculated by deseasonalising and detrending the time-series for each pixel for 1979–2016 (2004–2016 for CarboScope04). Positive anomalies correspond to a stronger-than-average CO_2_ sink or a below-average source.

The two inversions differ not only in aggregated global and tropical NBP_anom_ during in 2015/2016 (figure 1) but also in the spatial distribution of NBP_anom_ during both years (figure 2). CAMS produces a typical source anomaly in most of the tropics and Southern Hemisphere but a sink anomaly over the Amazon in both years, although the low density of the surface observations might not be sufficient to isolate the Amazon from the larger scale (figure 2*a*,*b*). In 2015, CarboScope04 reports negative NBP_anom_ evenly distributed over the tropics (excepting the Sahel), intensified in 2016 in Africa and Southeast Asia (figure 2*c*,*d*). The MMEM points to negative NBP_anom_ in the tropics, particularly in the Amazon and eastern Brazil, southern Africa and Australia (figure 2*e*,*f*). Generally, inversions and LSMs agree on a transition from weak to strong negative NBP_anom_ in southern Africa between 2015 and 2016 (figure 2; electronic supplementary material, figures S1 and S2). In the Amazon, the evolution of NBP_anom_ during 2015/2016 differs widely between LSMs, with some reporting negative anomalies (relative source) in both years (e.g. CLM4.5, VEGAS), others an anomalous source in 2015 followed by an anomalous sink in 2016 (e.g. ISAM, ORCHIDEE) or the inverse (JSBACH). Large differences in simulated NBP in 2015/2016 are also observed in central and southern Africa.

### (c) Seasonal evolution of NBP anomalies in 2015/2016

**Figure 3.**
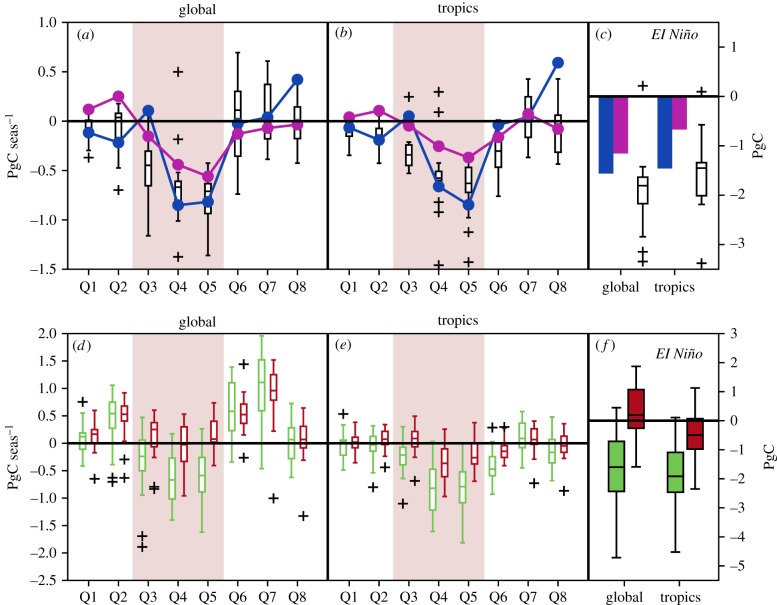
Evolution of carbon cycle anomalies during the 2015/2016 El Niño event. (*a–c*) Seasonal NBP_anom_ between January 2015 and December 2016 estimated by CAMS (dark blue) and CarboScope04 (magenta) and LSMs (boxplots indicate the model distribution) for the globe (*a*) and the tropics (*b*) and integrated values during El Niño, i.e. the sum of anomalies during Q3–Q5, indicated by the light red-shades ((*c*), bars for inversions and boxplots LSMs). (*d–f*): seasonal GPP_anom_ (green) and TER_anom_ (red) for the globe (*d*) and tropics (*e*) from LSMs during 2015–2016 and integrated during El Niño (*f*). The boxplots show the inter-quartile range (IQR) and median of anomalies estimated by LSMs, the whiskers the interval corresponding to 1.5 IQR and + markers indicate outliers.

Strong El Niño conditions started around May 2015, earlier than typical El Niño events, and ceased before the end of 2016. We analyse whether LSMs are able to capture the seasonal terrestrial sink response to the evolution of El Niño, compared to the two atmospheric inversions (figure 3*a–c*). We follow the approach by Yue *et al.* [19] and analyse consecutive trimesters over the 2 years. During January–March and April–June 2015 (Q1, Q2), inversions and the MMEM report close-to-average global and tropical sinks (anomalies below 0.2 PgC/season, negative for CAMS and LSMs, and positive for CarboScope04), consistent with pre-El Niño conditions. LSMs and inversions agree on the general decrease of the global and tropical C-sinks during the onset, peak and demise of El Niño from July–September 2015 (Q3) to April–June 2016 (Q6), but show differences in the exact timing and magnitude of anomalies.

Globally, CarboScope04 reports NBP_anom_ of −0.2 to −0.6PgC/season from Q3 until Q6, and CAMS reports large negative NBP_anom_ of −0.9 and −0.8 PgC/season in Q4 and Q5. Both inversions agree on the strong contribution of the tropics to the global NBP anomalies, 67% and 105% for CAMS (the value over 100% indicating a compensating effect from the extra-tropics) and 42–89% for CarboScope04. During the El Niño event (i.e. from Q3 to Q5, figure 3), CAMS and CarboScope04 report global integrated NBP_anom_ of −1.6 PgC and −1.2PgC (93% and 58% in the tropics, respectively), while MMEM estimates global NBP_anom_ of −1.8PgC (of which 83% in the tropics). Global C-sink anomalies during Q4–Q5 from LSMs are within the range of the two inversions with −0.7 PgC/season, but with a substantially more negative anomaly in Q3 (−0.4 PgC/season). These differences are mainly due to the larger negative anomalies at the onset of El Niño (in Q3) by LSMs compared to inversions.

Focusing on the tropics, LSMs show an earlier decrease in NBP_anom_ than inversions, with negative NBP_anom_ already in Q3. After Q3, LSMs and inversions show a remarkable agreement, with a peak negative NBP_anom_ occurring in January–March 2016 (Q5) then recovering and returning to neutral conditions by Q6 and Q7. In terms of magnitude, MMEM anomalies (−0.7 PgC/season and −0.8 PgC/season in Q4 and Q5, respectively) are between the two inversions, which report a decrease of NBP by 0.4–0.95 PgC/season in Q4 and by 0.6–0.8 PgC/season in Q5.

The overestimation of global NBP_anom_ in Q3 is mainly explained by the tropics, potentially due to too high fire emissions simulated by LSMs during the onset of the El Niño event. Fire emission anomalies from those models simulating fire (reported by only 7 out of 16 LSMs) (electronic supplementary material, figure S3) are indeed, on average, 0.2 PgC yr^−1^ and 0.3 PgC yr^−1^ higher than the annual anomalies of GFED4.1s in 2015 and 2016, respectively. This overestimation probably occurs in Q3 and Q4, when models report very high fire emissions, and consequently, stronger negative NBP_anom_ (−0.7 PgC/season for models with fire, compared to −0.4 PgC/season for other models in Q3). In Q4, anomalies in the tropics from LSMs are closer to the lower value of CAMS.

### (d) Driving processes

For further insight into the processes driving the land sink response to El Niño, we analyse the seasonal evolution of GPP_anom_ and TER_anom_ simulated by the LSMs (figure 3*d–f*) during 2015/2016. Electronic supplementary material, figure S4 additionally shows spatial GPP_anom_ estimated by the MMEM from Q1 to Q8. LSMs indicate an increase in GPP during the first half of 2015 mainly in the extra tropics (consistent with the record greening that year [19,62]). Only a few regions in southern Africa and the Sahel and in Australia registered negative GPP_anom_ already in Q1 and Q2 (electronic supplementary material, figure S3). The MMEM shows negative global GPP_anom_ during the abrupt onset of El Niño (Q3), but also large spread, while negative GPP_anom_ and spread in the tropics are still relatively small for Q3. Most LSMs estimate a strong negative global and tropical GPP_anom_ during the peak of El Niño (Q4 and Q5), mostly over the Amazon and eastern Brazil, as well as extra-tropical southern Africa and Australian regions (electronic supplementary material, figure S4). LSMs simulate weak negative GPP_anom_ in India and Southeast Asia. The sharp recovery in Q6 and Q7 is seen in global GPP, but not yet in the tropics, as GPP in northern South-America, southern Africa, northern Australia and Southeast Asia remains below average. The MMEM indicates positive global TER_anom_ (causing a greater source or lower sink) during both years and in particular near the end of the El Niño event (Q6 and Q7). However, in the tropics, TER decreases in phase with GPP (but with smaller magnitude) during the entire El Niño event, dropping in Q4 and Q5 and recovering in Q6 and Q7. During the peak of El Niño, MMEM shows strong negative or close to neutral TER_anom_ over most of the tropics (electronic supplementary material, figure S5), except for central Africa (where above-average GPP is simulated). The spatio-temporal evolution of simulated TER_anom_ appears, thus, to be mainly dominated by changes in GPP.

The spatio-temporal evolution of simulated GPP_anom_ mentioned above followed the progressive drying as El Niño developed (evaluated using a multi-scalar drought index at 6-month time-scale; electronic supplementary material, figure S6). The peak of El Niño in Q4 and Q5 corresponded to increasing intensity and spatial extent of drought conditions, affecting almost all tropical regions in South America, Asia and Australia and persisting until Q6 or even Q7 (South America and Australia). Even though in South America the peak of drought coincided with widespread negative GPP_anom_, the largest decreases in productivity are observed in typically dry regions, while humid areas (central Amazon) show smaller anomalies in productivity and recover faster (with positive anomalies in Q7). In Africa, the dipole of wet conditions in central tropics versus strong dryness in the south largely matches that of GPP_anom_.

### (e) Comparison with satellite-based data

**Figure 4.**
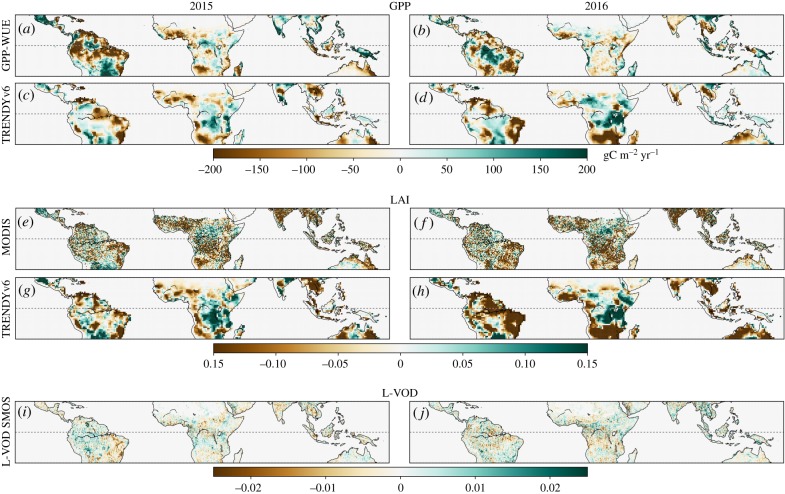
Comparison with observation-based datasets during 2015 and 2016 ((*a,c,e,g,i*) and (*b,d,f,h,j*), respectively) over the tropics (23°S–23°N). Spatial patterns of satellite-based LAI_anom_ from MODIS C6 (2000–2016) and modelled LAI anomalies from the LSM MMEM (*a–d*, 12 out of 16 models). GPP_anom_ calculated using a water-use efficiency model and remote-sensing data (GPP-WUE, 2000–2016) and GPP_anom_ simulated by the MMEM (*e–h*). Temporal changes in L-VOD over each year (*i,j*).

We evaluate whether simulated anomalies in vegetation status and productivity are consistent with LAI from MODIS and GPP derived from satellite data using a water-use efficiency model (GPP-WUE), shown in figure 4. We further evaluate changes in vegetation-optical depth as a proxy for changes in aboveground biomass. LSMs estimate widespread negative LAI anomalies in most of the tropics in both years, consistent with MODIS LAI. LSMs simulate positive LAI_anom_ for the humid forests in Africa, where MODIS LAI_anom_ shows more heterogeneity. Both MODIS and simulated LAI report an amplification of negative anomalies in 2016, also extending to parts of the Amazon.

The regions with strongest LAI decrease roughly coincide with those regions where below-average anomalies are found in both WUE-derived and simulated GPP: dry forests in tropical South America, the southern section of Africa and the Sahel, continental Southeast Asia and northern Australia. The agreement between WUE-GPP_anom_ and MMEM GPP_anom_ is better in 2015 than in 2016, though. In humid forests in Africa, WUE-GPP shows generalized negative anomalies in 2016, while LSMs simulate positive GPP_anom_.

The L-VOD index used here is more sensitive to the whole vegetation layer than other indices, which are more sensitive to the upper part of the canopy [59]. Even though L-VOD decrease (biomass reduction) is registered in the dry forests and savannahs of South America as in LAI and GPP, positive L-VOD changes (i.e. biomass accumulation) are observed in regions with negative LAI and WUE-GPP_anom_, e.g. India and Southeast Asia in 2016. This might indicate areas where vegetation is more resilient to the drought and appears to be more consistent with LSM and inversion estimates (figure 2). In the Amazon, on the other hand, L-VOD indicates a mixed pattern of negative and positive changes during 2015 and positive during 2016, while LSMs present predominantly negative GPP_anom_ and NBP_anom_ (figures 2 and 4).

## 4. Discussion

Our results show that the LSMs in TRENDYv6 can reproduce IAV patterns of the global terrestrial C-sink very close to the anomaly in the residual sink from GCB2017 and within the spread of atmospheric transport model inversions. The two inversions differ by up to 0.5 PgC yr^−1^ in particular years, especially in the tropics during El Niño events (e.g. 1997 and 2015). NBP from LSMs captures the general response of the carbon cycle to El Niño globally and over the tropics, but the agreement with inversions depends on the particular event considered. In 2015/2016, LSMs and inversions consistently estimate a decrease in terrestrial C uptake (2.0 PgC for MMEM, 1.5 PgC in 2015/2016 for CAMS and 1.0 PgC for CarboScope04), but smaller than the Global Carbon Budget estimate (3PgC in the 2 years, table 1).

At the seasonal scale, the LSMs simulate peak decrease in NBP in the late 2015 and early 2016 (Q3 to Q5), consistent with anomalies reported by inversions (figure 3). These results are also in line with observations of total column CO_2_ from OCO-2 [63] that show an increase in tropical CO_2_ concentrations from August 2015 onwards, in response to increased fire emissions and reduced terrestrial CO_2_ uptake.

LSMs point to the generalized decrease in tropical GPP at the end of 2015 and persisting until mid-2016 contributing the most to tropical NBP_anom_. The spatial patterns of LAI_anom_ and GPP_anom_ in 2015/2016 estimated by the MMEM are in good agreement with MODIS LAI and WUE-GPP_anom_, adding confidence to the simulated results, but are partly in contradiction to a recent study by Liu *et al.* [21]. Liu *et al.* contrast 2015 with 2011 (a La-Niña year associated with record breaking land-sink [11]), while we report anomalies relative to the 1979–2016. Nevertheless, their estimates of tropical CO_2_ anomalies in 2015 are still high even if we use 2011 as a reference to calculate inversion and LSM anomalies: −1.6 PgC yr^−1^, −0.7 PgC yr^−1^ and −1.9 PgC yr^−1^ for CAMS, CarboScope04 and MMEM, respectively. That study pointed to distinct continental-scale processes explaining anomalies in CO_2_ fluxes: GPP decrease in tropical America, TER increase in Africa and fire activity in Asia. Our results agree on the dominant role of GPP decrease in South America during El Niño. However, we find strong intra-continental heterogeneity, with strongest negative GPP_anom_ in dry forests and savannahs, consistent with previous studies showing the dominant role of semi-arid ecosystems in controlling carbon cycle sensitivity to ENSO [3,4]. Neither study does a perfect attribution of TER: TER in [21] is calculated as a residual term and might therefore be affected by errors in their NBP, GPP and fire emission estimates; at the same time, LSMs do not represent realistically the sensitivity of TER to precipitation [64]. Contrary to [21], LSMs indicate that tropical TER also decreased overall, probably because of the reduced substrate of TER or inhibition of decomposition due to drought. In Africa, the LSMs simulate a dipolar pattern during the peak of El Niño for both GPP_anom_ and TER, with an increase in the 0°–20°S region but a decrease in both variables further south. WUE-GPP shows similar results for 2015, but points to generalized negative GPP_anom_ in 2016. The decrease in TER in regions with decreased GPP may indicate a strong coupling of TER with biomass production in LSMs, as spatio-temporal anomalies in GPP and TER are mainly in phase, as noted previously [5].

The subset of LSMs that simulate fires shows a moderate increase in emissions (global average of 0.2 GtC for the 2 years), but significantly lower than the Liu *et al.* [21] estimate of fire emission increase of 0.4 GtC for South Asia only. This difference may be due to the lack of peat fires in LSMs but is hard to reconcile with the lower GFED4.1s estimate of global fire emissions (electronic supplementary material, figure S3). LSMs could show too little sensitivity of TER and fires to climate variability, several models sharing similar parametrizations to represent soil decomposition response to temperature and water stress for example. Conversely, the Liu *et al.* [21] study uses sun-induced chlorophyll fluorescence as an indirect measure of GPP and carbon monoxide (CO) concentrations as a proxy for fires. How these relationships or systematic errors in assimilated total column CO_2_ retrievals vary between normal and El Niño years is still unclear.

Even though below-average GPP was registered in the Amazon (especially in 2016) in both LSM simulations and WUE-GPP, the strongest decreases in GPP occur in the tropical dry forest and savannahs in South America, southern Africa and northern Australia. This points to a predominant role of water availability in the observed response to the 2015/2016 El Niño and is consistent with previous studies [3,4,9,39,59]. Indeed, the spatio-temporal evolution of simulated tropical GPP decrease during the onset and peak of the 2015/2016 El Niño follows the progressive increase in dryness (electronic supplementary material, figure S6). Additionally, LSMs indicate that dry forests and semi-arid biomes respond more strongly than humid ones to similar drought conditions and also point to a faster recovery of the humid Amazon forest in the second half of 2016 (electronic supplementary material, figure S4), when drought conditions started to become more moderate (electronic supplementary material, figure S6).

It is worth pointing out that the good agreement between LSMs and inversions or satellite-based observations is especially true for the MMEM, while individual models may show substantially different regional response over the course of the 2015/2016 event, although most individual LSMs show anomalies consistent with the MMEM across the tropics (electronic supplementary material, figure S7). Since all models use the same climate and land-use forcing, the differences in model responses arise because of the different parametrizations of carbon cycle processes, resulting in different model sensitivities to the increasingly warm and dry conditions observed until the peak of El Niño. The added value of using MMEM is recognized Earth system modelling, and several examples exist of applications in which combined information from several models is superior to results from any single model [65]. In the climate community, the diversity amongst models is considered a healthy aspect and provides a basis for estimating uncertainty [66].

## 5. Conclusion

We show that the LSM ensemble reproduces the spatial and temporal impacts of the 2015/2016 El Niño on the terrestrial C-sink within the inversions' range. We find that the decrease in the global terrestrial sink during El Niño in 2015/2016 can be mainly explained by decreased tropical GPP, in response to the ENSO-related drought in transitional to semi-arid regions, with a secondary role of the increase in fires and ecosystem respiration. It is still unclear whether TER plays an important role in controlling NBP_anom_ during El Niño events. Our results agree with recent work highlighting the control of NBP by water availability [3,5]. However, this agreement might be ENSO event-dependent, as we found larger disagreement between inversions and LSMs in 1997/1998 than in 2015/2016. Understanding how terrestrial biogeochemical processes contribute to the emergent response of ecosystems to warming and drying during El Niño events is crucial to comprehend the vulnerability of land ecosystems to future changes in climate in the tropics and other sensitive regions.

## Supplementary Material

Supplementary Datasets

## Supplementary Material

Supplementary Figures

## Data accessibility

CO_2_ fluxes from the CAMS atmospheric inversion are freely available at http://atmosphere.copernicus.eu/. CarboScope datasets are available at http://www.bgc-jena.mpg.de/CarboScope/. The monthly time series of global and tropical CO_2_ fluxes are provided for each inversion in electronic supplementary material. Outputs from land surface models from the TRENDYv6 project used in this study are provided in electronic supplementary material. These are the time series of global/tropical average monthly NBP, GPP, TER and fire emissions from each individual model, as well as the annual and seasonal gridded anomalies of the MMEM NBP, GPP, TER and LAI. Annual and seasonal anomaly NBP maps from inversions, and annual NBP maps from individual models are also provided. The full TRENDYv6 dataset including other outputs is available, subject to the individual modelling groups' agreement, via a request to S. Sitch (s.a.sitch@exeter.ac.uk). The results from the Global Carbon Budget 2017 are available for download at http://www.globalcarbonproject.org/carbonbudget/17/data.htm. Annual anomalies of WUE-GPP are provided in electronic supplementary material. The GFED4.1s fire emission database is publicly available at http://www.globalfiredata.org/data.html. MODIS data are freely available at https://lpdaac.usgs.gov/dataset_discovery/modis/modis_products_table. The L-VOD data can be accessed at the Centre Aval de Traitement des Données SMOS (CATDS): ftp://ext-catds-cecsm:catds2010@ftp.ifremer.fr/Land_products/L3_SMOS_IC_Soil_Moisture/Seasonal_Averages/. The Standardized Precipitation-Evapotranspiration Index is freely available at http://spei.csic.es/.
