# Supplementary material for: Impact of the 2015/2016 El Niño on the terrestrial carbon cycle constrained by bottom-up and top-down approaches
Source: Philos Trans R Soc Lond B Biol Sci. 2018 Oct 8;373(1760):20170304. doi: 10.1098/rstb.2017.0304 (PMC6178442; doi:10.1098/rstb.2017.0304)
Supplement: Supplementary Figures [file rstb20170304supp2.zip › FS1.pdf]

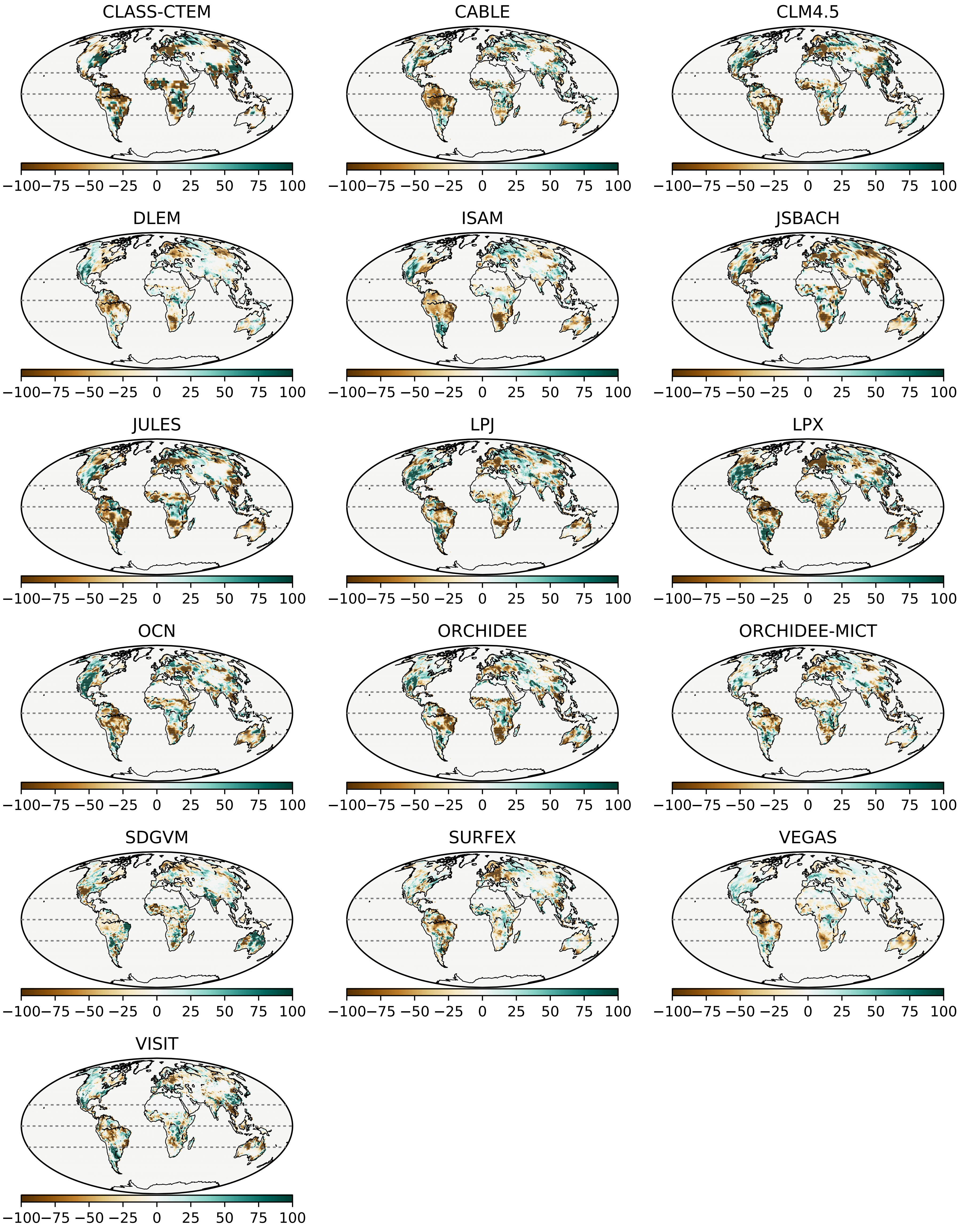
CLASS-CTEM
−100 −75 −50 −25 0 25 50 75 100
CABLE
−100 −75 −50 −25 0 25 50 75 100
CLM4.5
−100 −75 −50 −25 0 25 50 75 100
DLEM
−100 −75 −50 −25 0 25 50 75 100
ISAM
−100 −75 −50 −25 0 25 50 75 100
JSBACH
−100 −75 −50 −25 0 25 50 75 100
JULES
−100 −75 −50 −25 0 25 50 75 100
LPJ
−100 −75 −50 −25 0 25 50 75 100
LPX
−100 −75 −50 −25 0 25 50 75 100
OCN
−100 −75 −50 −25 0 25 50 75 100
ORCHIDEE
−100 −75 −50 −25 0 25 50 75 100
ORCHIDEE-MICT
−100 −75 −50 −25 0 25 50 75 100
SDGVM
−100 −75 −50 −25 0 25 50 75 100
SURFEX
−100 −75 −50 −25 0 25 50 75 100
VEGAS
−100 −75 −50 −25 0 25 50 75 100
VISIT
−100 −75 −50 −25 0 25 50 75 100
