# Supplementary material for: Impact of the 2015/2016 El Niño on the terrestrial carbon cycle constrained by bottom-up and top-down approaches
Source: Philos Trans R Soc Lond B Biol Sci. 2018 Oct 8;373(1760):20170304. doi: 10.1098/rstb.2017.0304 (PMC6178442; doi:10.1098/rstb.2017.0304)
Supplement: Supplementary Figures [file rstb20170304supp2.zip › FS2.pdf]

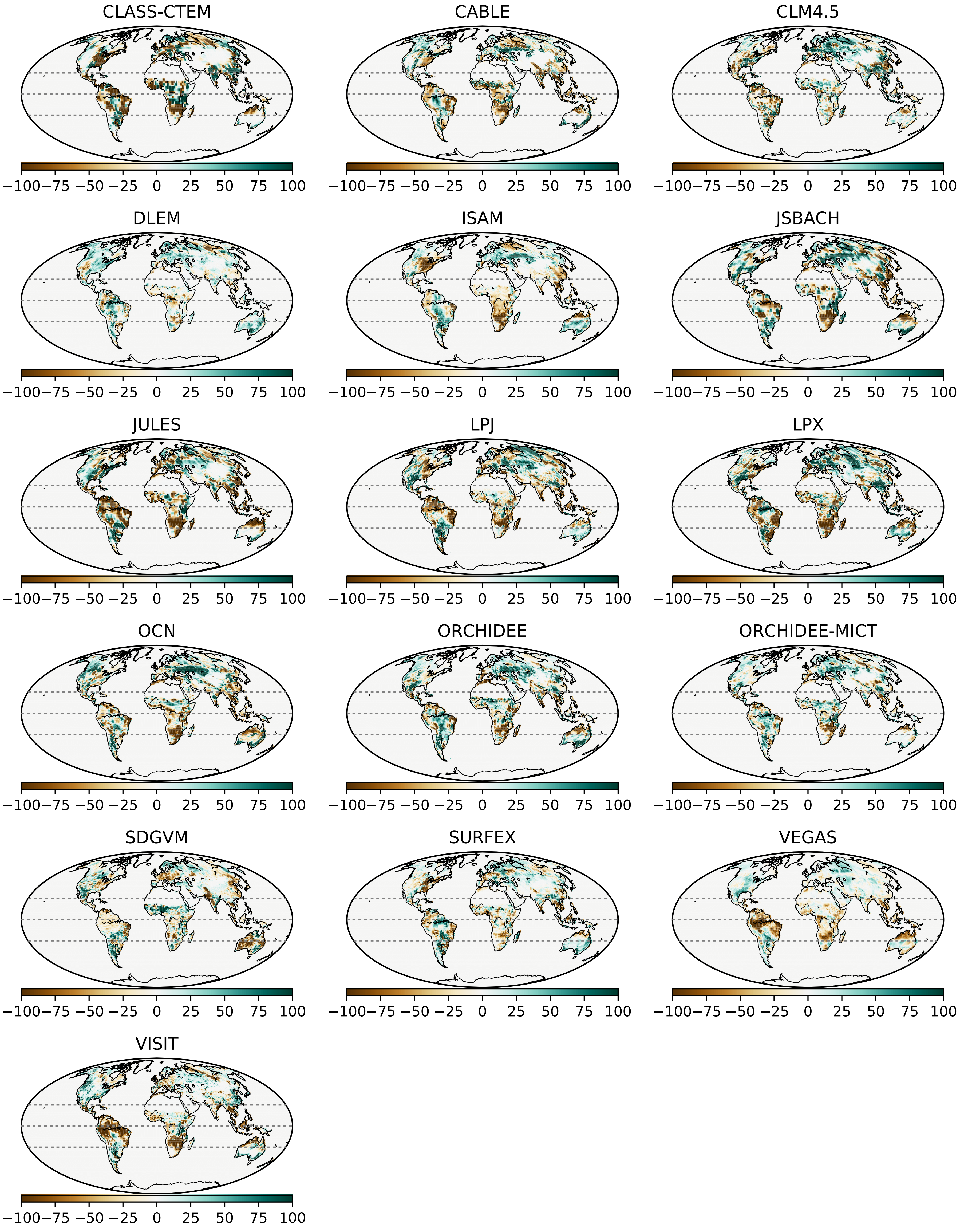
CLASS-CTEM
CABLE
CLM4.5
DLEM
ISAM
JSBACH
JULES
LPJ
LPX
OCN
ORCHIDEE
ORCHIDEE-MICT
SDGVM
SURFEX
VEGAS
VISIT
−100 −75 −50 −25 0 25 50 75 100
