# Supplementary material for: Impact of the 2015/2016 El Niño on the terrestrial carbon cycle constrained by bottom-up and top-down approaches
Source: Philos Trans R Soc Lond B Biol Sci. 2018 Oct 8;373(1760):20170304. doi: 10.1098/rstb.2017.0304 (PMC6178442; doi:10.1098/rstb.2017.0304)
Supplement: Supplementary Figures [file rstb20170304supp2.zip › Supplementary Figure captions.docx]

Supplementary Figures

Figure S1. NBPanom from individual TRENDYv6 models during 2015. The negative values correspond to lower (higher) than average sink (source) and positive anomalies correspond to increased (decreased) sink (source).

Figure S2. NBPanom from individual TRENDYv6 models during 2016. The negative values correspond to lower (higher) than average sink (source) and positive anomalies correspond to increased (decreased) sink (source).

Figure S3. Carbon emission anomalies from fires simulated by the LSMs (brown lines, 1979-2016) and reported by the GFED4.1s (black line, 1997-2016) database (left panel). Only 7 out of 16 models provided monthly values of fire emissions (Table 2, models with annual values not used), which were used to assess the seasonal evolution of simulated anomalies in fire emissions during 2015/16 (right panel).

Figure S4. Seasonal evolution of tropical GPP, estimated by the LSM MMEM. The negative values correspond to lower (higher) than average productivity (source) and positive anomalies correspond to increased (decreased) productivity (source).

Figure S5. Seasonal evolution of tropical TER, estimated by the LSM MMEM. The colorbar follows the convention of the corresponding impact on NBPanom: negative TERanom increase the sink and are therefore represented in green, while positive TERanom contribute to a negative NBPanom and are therefore represented in brown.

Figure S6. Seasonal evolution of the multi-scalar Standardised Precipitation-Evapotranspiration Index (<http://spei.csic.es/)>. Contrary to other drought indices SPEI takes into account not only deficits in precipitation, but also in evaporative demand. The SPEI is calculated in standard-deviation units relative to the climatological values at different temporal scales ranging from 1 month to 24 months. Here we used the monthly SPEI index at 6-month time-scales (i.e. for a given month m, SPEI06 indicates dryness accumulated from m-5 to m) between 1979-2016 and spatial resolution of 0.5^o^×0.5^o^. SPEI values were deseasonalised and detrended relative to the 1979-2016 average.

Figure S7. Number of models reporting positive (purple) or negative (brown) annual anomalies of NBP in 2015 and 2016 respectively.
