# Supplementary figures and images for: Impact of the 2015/2016 El Niño on the terrestrial carbon cycle constrained by bottom-up and top-down approaches
Source: Philos Trans R Soc Lond B Biol Sci. 2018 Oct 8;373(1760):20170304. doi: 10.1098/rstb.2017.0304 (PMC6178442; doi:10.1098/rstb.2017.0304)

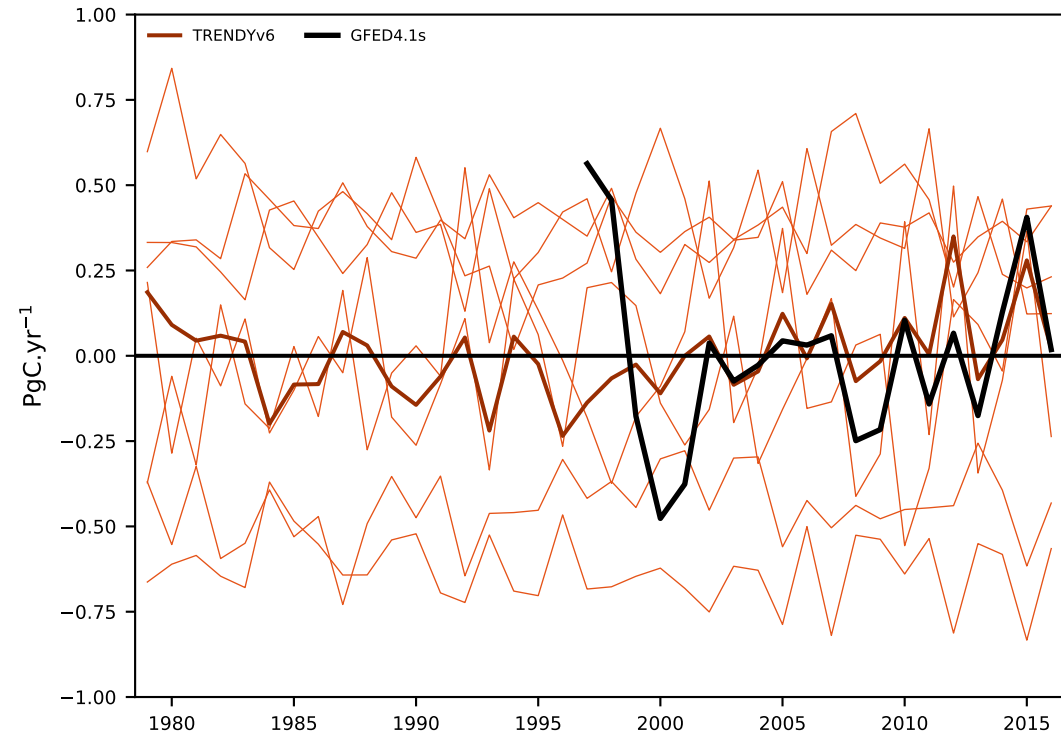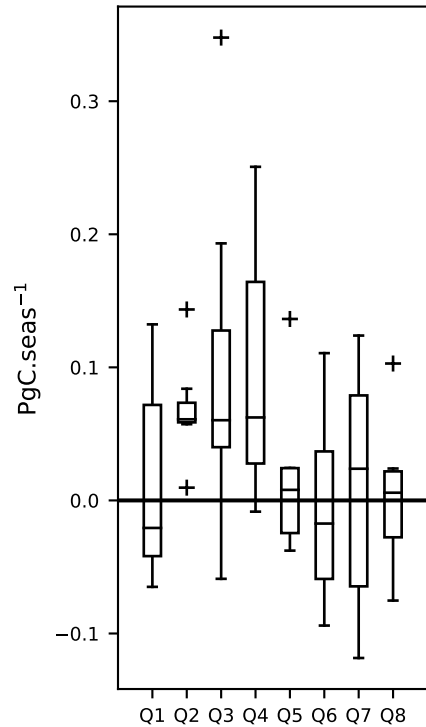

Supplement: Supplementary Figures [file rstb20170304supp2.zip › FS3.pdf]

## GPP

2015

2016

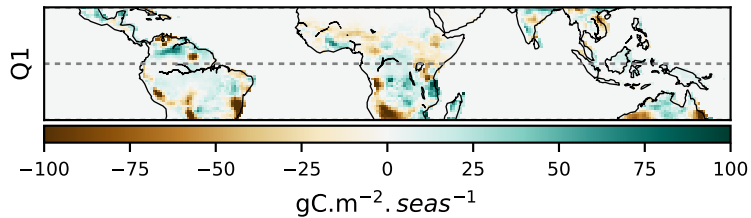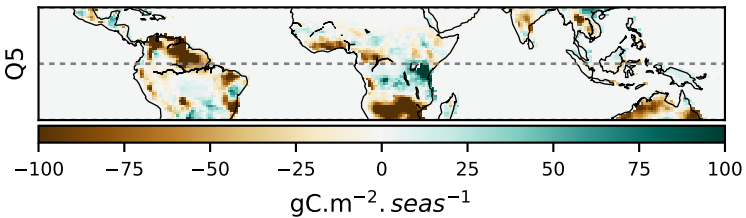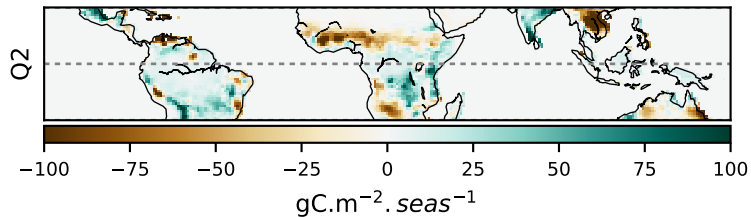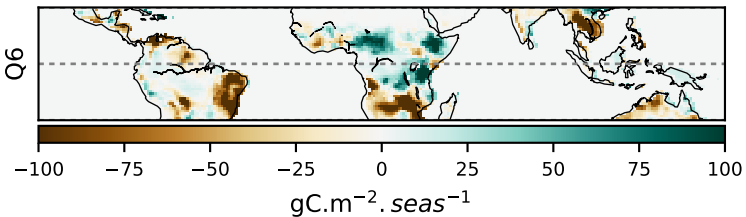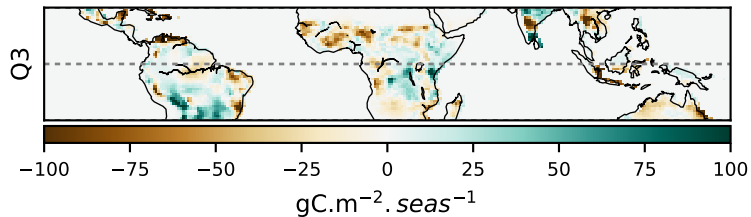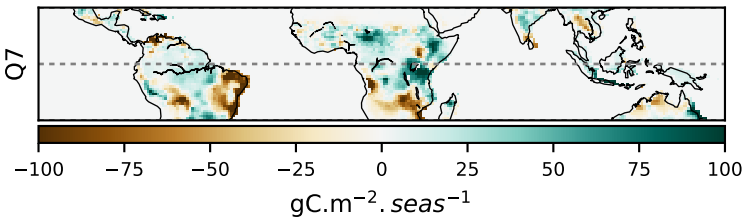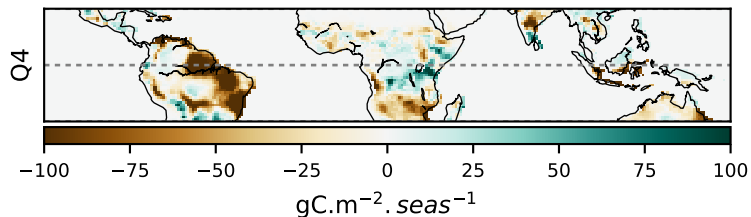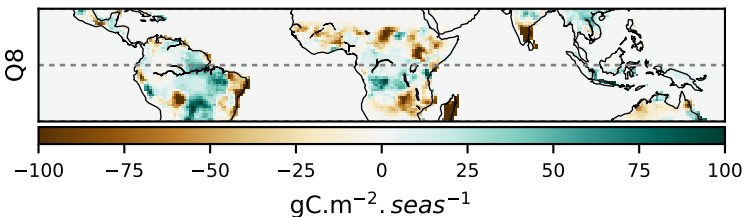

Supplement: Supplementary Figures [file rstb20170304supp2.zip › FS4.pdf]

TER

2015

2016

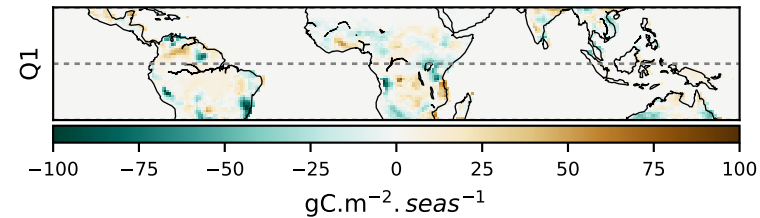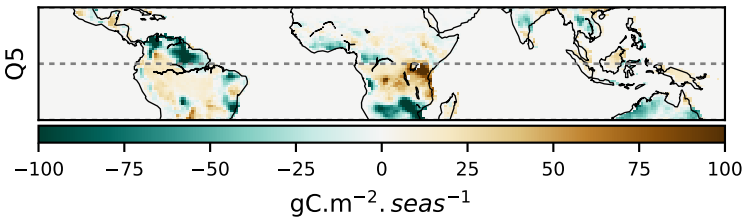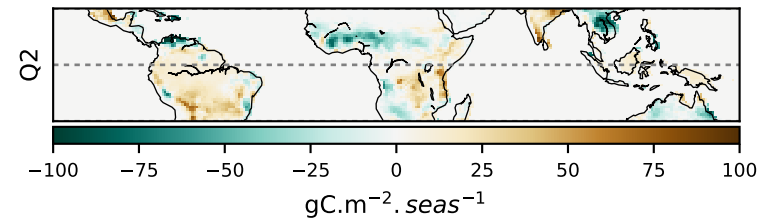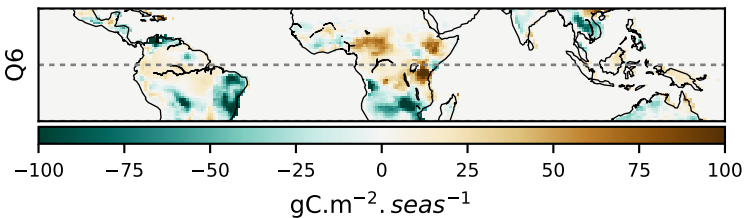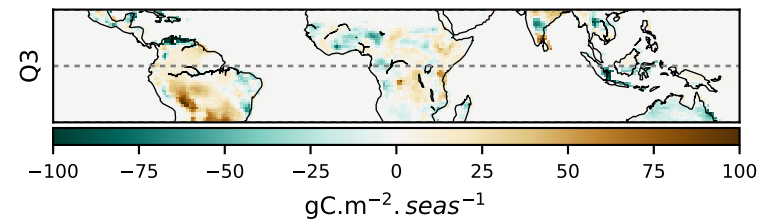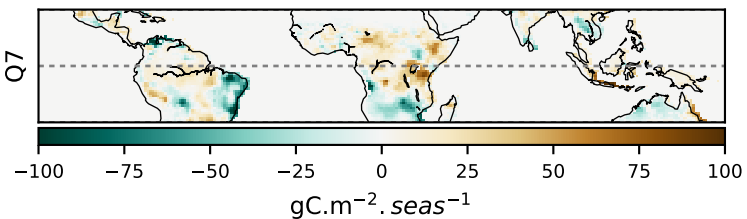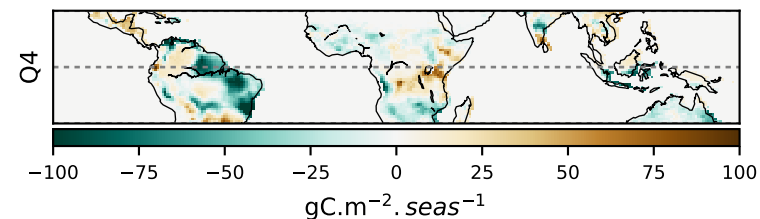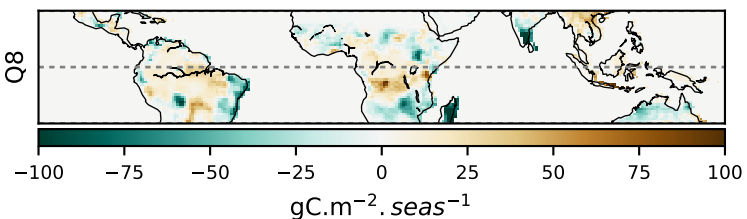

Supplement: Supplementary Figures [file rstb20170304supp2.zip › FS5.pdf]

## SPEI 6M

2015

2016

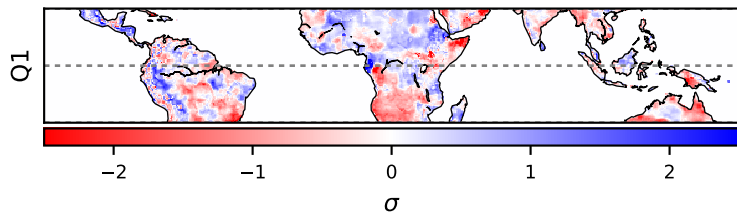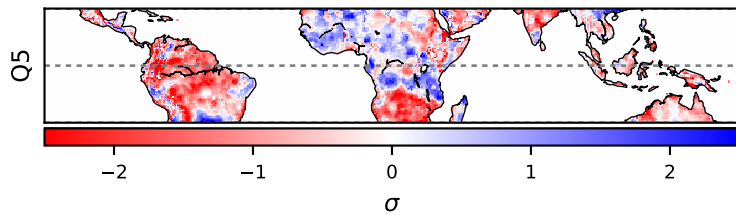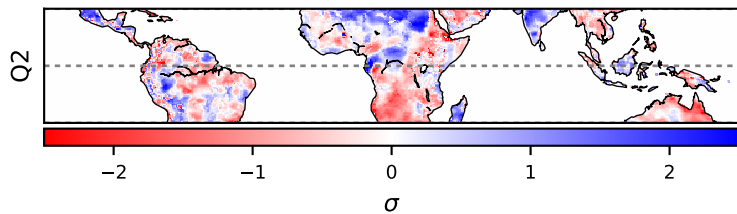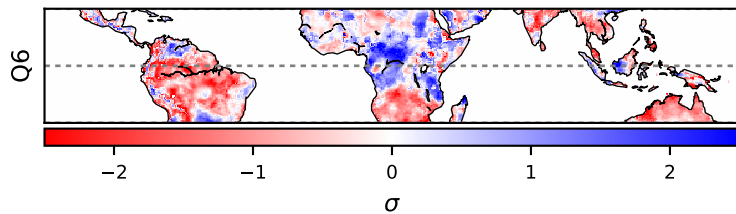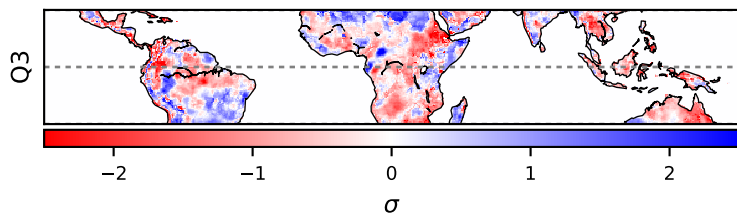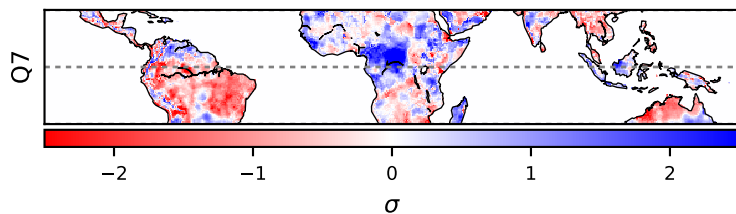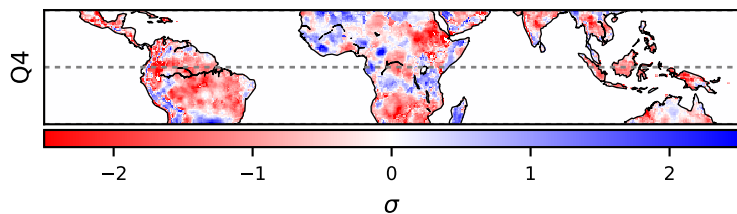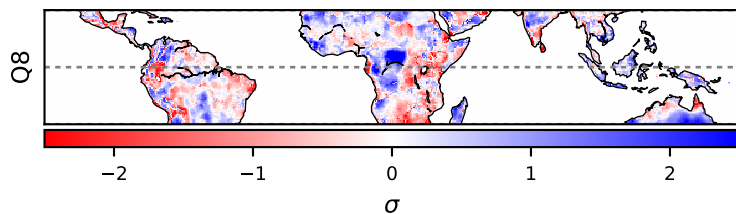

Supplement: Supplementary Figures [file rstb20170304supp2.zip › FS6.pdf]

2015

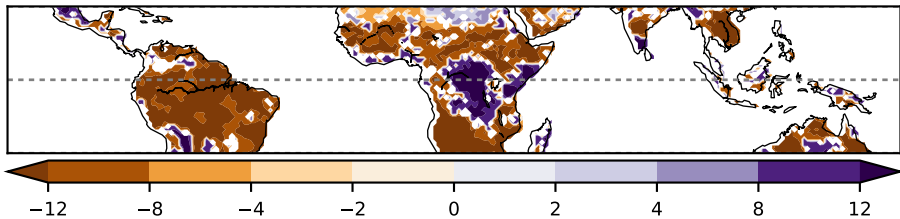

N models

2016

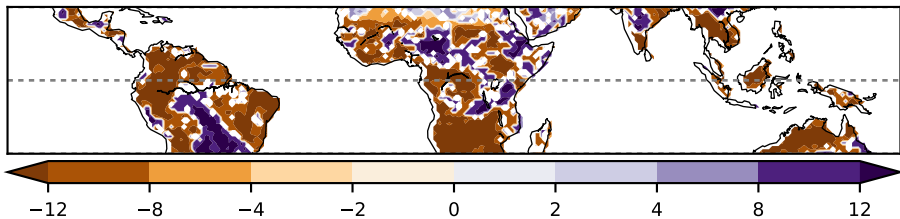

N models

Supplement: Supplementary Figures [file rstb20170304supp2.zip › FS7.pdf]
